# Appropriateness of the EQ-5D-5L in capturing health-related quality of life in individuals with transfusion-dependent β-thalassemia: a mixed methods study

**DOI:** 10.1186/s12955-024-02265-8

**Published:** 2024-07-11

**Authors:** Adriana Boateng-Kuffour, Hanna Skrobanski, Jennifer Drahos, Puja Kohli, Katie Forster, Sarah Acaster, Zahra Pakbaz, Nanxin Li, Kate Williams

**Affiliations:** 1 Vertex Pharmaceuticals, Boston, MA USA; 2 Acaster Lloyd Consulting, London, UK; 3 Division of Hematology Oncology, University of California Irvine School of Medicine, Orange, CA USA; 4 Health Economics and Outcomes Research, Vertex Pharmaceuticals Incorporated, 50 Northern Avenue, Boston, MA 02210 USA

**Keywords:** Transfusion-dependent β-thalassemia, Content validity, EQ-5D-5L DS, Health state utility, Health-related quality of life, Mixed methods analysis

## Abstract

**Background:**

Individuals with transfusion-dependent β-thalassemia (TDT) experience symptoms and functional impacts that reduce their health-related quality of life. However, EQ-5D-derived health utility index scores in TDT often indicate good HRQoL, suggesting the EQ-5D may not adequately capture the impact of TDT. This study explored the disease and treatment burden of TDT and examined the appropriateness of the EQ-5D-5L descriptive system (DS) in measuring HRQoL in TDT.

**Methods:**

Adults with TDT in the United Kingdom, United States, and France completed a background questionnaire and EQ-5D-5L DS, followed by 60-minute semi-structured interviews on symptoms and HRQoL impacts of TDT (concept elicitation) and appropriateness of EQ-5D-5L DS (cognitive debrief). Transcribed interviews were analyzed using thematic and content analyses. The relationship between TDT symptoms and impacts were summarized in a conceptual model. EQ-5D-5L DS was mapped to concepts identified in the qualitative data to assess its capture of HRQoL concepts. Participants’ EQ-5D-5L DS scores were compared to their qualitative descriptions for each dimension to assess their concordance.

**Results:**

Thirty participants in the United States (*n* = 14 [46.7%]), United Kingdom. (*n* = 12 [40.0%]), and France (*n* = 4 [13.3%]) completed the study (73.3% female; mean age = 28.4 years [standard deviation (SD) = 5.1]; mean annual red blood cell transfusion [RBCT] frequency = 18.4 [SD = 7.6]). Participants reported TDT symptoms and impacts on HRQoL, all fluctuating across the RBCT cycle. EQ-5D-5L DS did not fully capture 11 of 16 (68.8%) HRQoL concepts reported. Most participants (*n* = 20/27 [74.1%]) reported that EQ-5D-5L DS did not capture important aspects of living with TDT, and 42.9% (*n* = 12/28) reported negative/neutral overall impressions of EQ-5D-5L DS. The highest degree of discordance between participants’ qualitative data and EQ-5D-5L DS dimension scores was observed with mobility (42.3%) and self-care (34.6%), where the qualitative descriptions relating to these dimensions were worse than their quantitative scores.

**Conclusion:**

Current findings suggest that EQ-5D-5L DS lacks content validity and the derived health utility index score may not fully represent the burden of disease in TDT.

**Supplementary Information:**

The online version contains supplementary material available at 10.1186/s12955-024-02265-8.

## Background

Transfusion-dependent β-thalassemia (TDT) is a hereditary anemia and progressive chronic disease often diagnosed within the first 2 years of life and characterized by ineffective red blood cell (RBC) production in bone marrow [1]. TDT arises from mutations in the β-globin gene, which codes for oxygen-carrying hemoglobin molecules in RBCs [1]. Individuals with TDT depend on regular RBC transfusions (RBCTs) for survival, leading to the accumulation of iron, which can result in progressive organ damage and life-threatening dysfunction [2]. Accordingly, patients require chronic iron chelation therapy (ICT) as part of TDT disease management [3]. Comorbidities from TDT and its treatments include heart failure, arrhythmia, diabetes, hypogonadism, hypothyroidism, chronic viral infections (HIV and hepatitis B and C), and thrombosis [1, 2]. The symptoms of TDT, along with its treatments and co-morbidities, is thought to impact the health-related quality of life (HRQoL) of individuals with TDT [4–7].

Generic preference-based measures are commonly used in clinical trials to assess HRQoL and estimate health utility values for use in cost-effectiveness analyses. One such measure commonly used for this purpose is the EQ-5D questionnaire [8, 9]. The National Institute for Health and Care Excellence in England and Wales specifies a preference for using the EQ-5D to measure HRQoL to maintain “consistency” across health technology appraisals [10]. It includes a descriptive system (DS) that assesses five dimensions of health: mobility, self-care, usual activities, pain/discomfort, and anxiety/depression. Responses are used to generate health state utility index scores, which are used to calculate quality-adjusted life-years to quantify the cost-effectiveness of new treatments [11, 12].The EQ-5D has both a three-level (3 L) and five-level (5 L) version. The 5 L version was developed to enhance sensitivity and reduce ceiling effects present within the 3 L version, which has been validated in multiple studies [12–14].

Findings from clinical trials and observational studies in TDT show high baseline EQ-5D-3L health utility index scores [15–17] similar to general population scores, suggesting good HRQoL [18–21]. However, some evidence suggests that either version of the EQ-5D can result in measurement error or a lack of sensitivity when used in other chronic health conditions [22–27]. For example, in a clinical trial for cystic fibrosis, participants with mild and severe lung dysfunction reported baseline health utility scores of 0.923 and 0.870, respectively [28], which are both higher than the population norms for the United Kingdom (0.856) and United States (0.867) [29]. To address this lack of sensitivity in certain disease states, ‘bolt-ons’ have been developed to assess additional dimensions that are specific to a health problem, which are appended to the generic instrument. However, these may still not cover all concepts relevant to TDT.

When EQ-5D evidence is not available or does not appropriately capture HRQoL in a particular patient population, the National Institute for Health and Care Excellence suggests providing qualitative and quantitative empirical evidence showing that key dimensions of health are not captured by the EQ-5D-5L DS [30]. Qualitative research allows for an in-depth exploration of the impact of a disease from the patient perspective, which can generate rich data and highlight symptoms and impacts on HRQoL that were unknown or undervalued. This type of research is particularly important in rare diseases such as TDT, where there is limited knowledge of the disease and a small pool of potential participants. Although some studies have explored the humanistic burden of TDT’s symptoms and conventional therapies [4, 15–17, 31–50], there is a dearth of qualitative research in this population.

There are also limited qualitative empirical data exploring the appropriateness of the EQ-5D-5L DS in assessing HRQoL in TDT. Additionally, the content validity of the EQ-5D-5L DS in TDT has not been formally evaluated. Mixed methods research, using qualitative and quantitative methods, is useful for exploring the potential sensitivity and validity of HRQoL instruments [51]. As such, this study used a mixed methods approach to explore the disease and treatment burden of TDT on individuals with this disease and examined the appropriateness of the EQ-5D-5L DS in measuring HRQoL in this population.

## Methods

### Overview of study design

In this mixed methods study, the EQ-5D-5L DS was administered to participants with TDT, who participated in 60-minute combined concept elicitation and cognitive debrief interviews. After participants completed a background questionnaire, including the EQ-5D-5L DS, semi-structured interviews were conducted to identify the patient-relevant impact of TDT on HRQoL (concept elicitation) and explore the relevance, comprehensiveness, and appropriateness of the EQ-5D-5L DS, including item stems, response options, and recall period (cognitive debriefing). This approach was adopted to examine whether the EQ-5D captures patient HRQoL concepts deemed critical to the lived experiences of TDT’s disease burden.

### Study participants

Participants were individuals with TDT living in the United States, United Kingdom, and France. The inclusion criteria were: (1) self-reported clinical diagnosis of TDT, (2) received eight or more RBCTs/year in each of the 2 years prior to enrollment, (3) aged 18 to 35 years (inclusive), (4) never undergone a hematopoietic stem cell transplant, received gene editing, and/or participated in a gene therapy clinical trial to treat TDT, and (5) willing and able to provide online informed consent to participate in the study.

### Study materials

Quantitative measures included the background questionnaire and EQ-5D-5L DS. The EQ-5D is a generic preference-based measure of HRQoL, comprising a five-item DS and EQ-visual analogue scale (EQ-VAS) [8]. The dimensions of health included are mobility, self-care, usual activities, pain/discomfort, and anxiety/depression. To complete the EQ-5D-5L DS, participants were asked to think about a day when their TDT was at its worst. “At its worst” was chosen as a time-point to ensure that participants were thinking about the same moment, as there was a time lag between their completion of the EQ-5D-5L and interview questions. The background questionnaire included questions about participants’ demographics (e.g., sex, age, employment status), disease/clinical characteristics (e.g., symptoms, complications due to transfusions and iron overload), and treatment (e.g., transfusion frequency, pain medication, ICT) for TDT.

Interviews were conducted according to a semi-structured interview guide, which was developed based on the published literature on the HRQoL of individuals with TDT [44–50], as well as consultation with TDT clinical experts. The first part of the interview guide included open-ended questions on the symptoms and HRQoL impacts of TDT and its treatment (concept elicitation). Questions were also included to elicit qualitative data related to each of the five EQ-5D-5L dimensions. The second part of the interviews included cognitive debrief questions to determine the perceived relevance of the EQ-5D-5L in measuring HRQoL in TDT.

### Ethics

The study was reviewed and approved by WIRB-Copernicus Group Independent Review Board (IRB tracking number: 20,217,056). All participants consented to the publication of their de-identified data.

### Recruitment and data collection

Participants were recruited by a third-party recruitment agency, who identified potential participants from their participant databases and via patient advocacy groups. All participants were provided with an information sheet outlining the study aims, processes, and participant responsibilities. All participants gave their written informed consent online to participate in the study. The interviews in the United States and United Kingdom were conducted in English by two study authors (HS and KF), both with an MSc or PhD in Psychology and over 12 years of combined qualitative research experience. The interviews in France were conducted by trained interviewers in French. None of the participants were known to the interviewers.

Qualitative interviews were conducted via teleconference between May and September 2022. At the start of each interview, participants re-confirmed their consent verbally. The interviews then followed the semi-structured interview guide and lasted approximately 1 h. Interviews were recorded and transcribed, then de-identified, and French-language transcripts were translated into English for analysis.

### Qualitative data analyses

All transcribed data were analyzed using MAXQDA qualitative analysis software [52]. Concept elicitation data were analyzed using thematic analysis to describe the reported symptoms of TDT and impacts of treatment for TDT on participants’ HRQoL. Thematic analysis is a qualitative method for identifying, analyzing, and reporting themes [53]. Each interview transcript was systematically coded on an initial coding framework and developed iteratively throughout the analysis.

Interviews were conducted until data saturation was attained, which is the point at which no new themes or concept were identified from additional qualitative data [54]. In order to plan the research, a minimum of 30 interviews was established to ensure a sufficient sample for the mixed methods analyses. This is consistent with qualitative research guidelines, recommending a broad estimate of between 20 and 30 interviews per cohort to achieve data saturation [55]. To assess whether data saturation had been achieved by 30 interviews, a saturation matrix was used to monitor the frequency of reported concepts across the interviews, with concepts listed in rows and participant numbers/country of residence listed in columns [56].

A conceptual model was developed to illustrate the experience of living with TDT and relationships between themes. The EQ-5D-5L DS was then mapped to the concepts identified in the qualitative data to identify any HRQoL TDT concepts not captured. Mapping involved the development of a data extraction matrix in Microsoft Excel [57] which ordered HRQoL interview concepts as columns and the five EQ-5D-5L DS dimensions as rows. Two study authors (HS and KF) independently verified whether each concept was covered (fully or partially) by at least one dimension, and any disrepancies were resolved through discussion. A third researcher (KW) reviewed and adjudicated any differences/disagreements between codes generated between the first two researchers. Cognitive debrief data were analyzed using qualitative content analysis, which provides a summary of responses to specific questions of interest. Data from the background questionnaire and EQ-5D-5L DS were summarized using descriptive statistics in Microsoft Excel [57].

### Mixed methods analyses

Participants’ qualitative descriptions relating to the EQ-5D-5L DS obtained from the concept elicitation data were compared to their quantitative EQ-5D-5L DS scores to assess the level of concordance between the two datasets. Data were deemed to be discordant if there was a clear difference between participants’ qualitative descriptions and EQ-5D-5L dimension scores (e.g., if a participant reported “no pain” on the EQ-5D-5L but reported experiencing pain during the interviews). Cases where data were ambiguous (e.g. if they reported slight problems on the EQ-5D-5L, but the qualitative description could be interpreted as slight or moderate) or missing were not included. Two study authors (HS and KF) independently verified the concordance or discordance of each participant qualitative and quantitative data. A third researcher (KW) reviewed and adjudicated any differences/disagreements between codes generated between the first two researchers.

## Results

### Participants’ self-reported demographics and clinical characteristics

Thirty participants were included in the study (United States: *n* = 14; United Kingdom: *n* = 12; France: *n* = 4). The mean age was 28.4 (SD = 5.1) years, and most were female (*n* = 22 [73.3%]). They received a mean of 18.0 (SD = 8.2) RBCTs in the previous year and 18.7 (SD = 7.7) RBCTs in the year before that. The key reported symptoms (≥ 50% frequency) present in the past 3 months included tiredness/fatigue (100%), weakness (90.0%), pain/discomfort (86.7%), shortness of breath (73.3%), and tachycardia (50.0%) (Table 1).

**Table 1:** Participants’ self-reported demographics and clinical characteristics

Characteristic	*N* = 30
**Country of residence**, *n* (%)	
United States	14 (46.7)
United Kingdom	12 (40.0)
France	4 (13.3)
Age (years), mean (SD)	28.4 (5.1)
**Gender**, ***n*(%)**	
Female	22 (73.3)
**Ethnicity**, ***n*(%)**	
Asian or Pacific Islander	18 (60.0)
Black or African American	1 (3.3)
White or Caucasian	5 (16.6)
Other	2 (6.6)
Not reported	4 (13.3)
**Education**, ***n*(%)**	
School/college-level qualification	4 (13.3)
Graduate degree or equivalent	13 (43.3)
Postgraduate degree or equivalent	9 (30.0)
Other formal qualification	4 (13.3)
Age at diagnosis (years), mean (SD)	1.1 (2.1)
Age at first blood transfusion (years), mean (SD)	2.2 (3.4)
Number of RBCTs in past year (0–1 year ago), mean (SD)	18.0 (8.2)
Number of RBCTs in the year before (1–2 years ago), mean (SD)	18.7 (7.7)
**TDT symptoms experienced in past 3 months**, ***n*(%)**	
Tiredness or fatigue	30 (100)
Weakness	27 (90.0)
Shortness of breath	22 (73.3)
Tachycardia (fast heartbeat)	15 (50.0)
Pain or discomfort	26 (86.7)

### Symptoms and impacts reported by participants

**Fig. 1:**
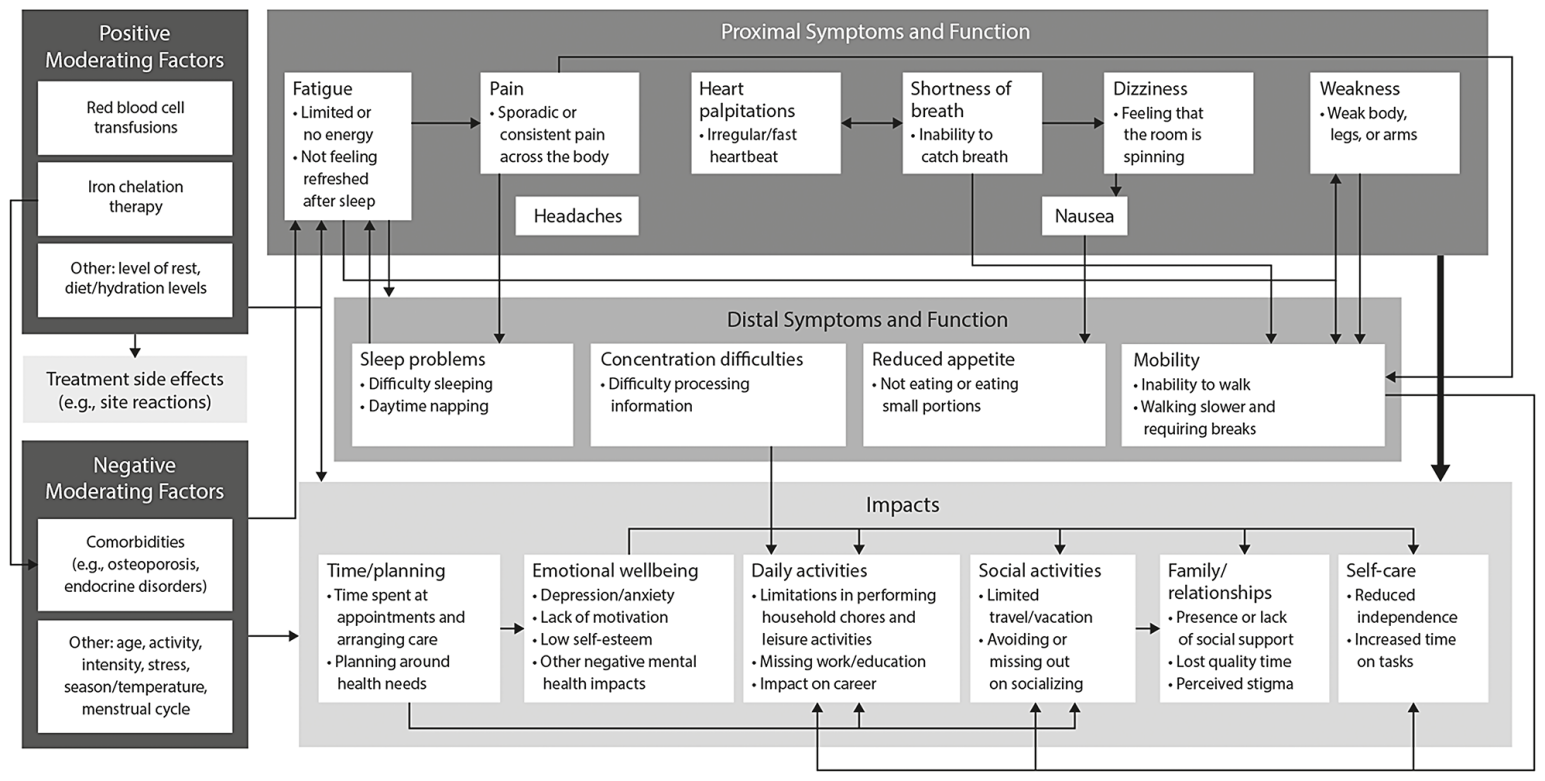
Conceptual model on the patient experience of TDT.*TDT* transfusion-dependent β-thalassemia

Figure 1 is a conceptual model illustrating the reported symptoms of TDT and its impacts on HRQoL, as well as the relationship between them and how they are affected by positive and negative moderating factors. The saturation matrix indicated that saturation was met for the broad overarching themes, with no new symptoms/functional issues emerging after four interviews and no new impacts on HRQoL emerging after two interviews (Additional File 1). Detailed qualitative analysis findings of TDT symptoms and impacts on HRQoL are provided in Additional File S2.

### Symptoms

Participants described experiencing a wide range of symptoms and functional issues, some that were a direct consequence of TDT (proximal symptoms) and others that were not (distal symptoms) (Fig. 1). Proximal symptoms included fatigue (*n* = 29), pain (*n* = 27), headaches (*n* = 17), shortness of breath (*n* = 17), heart palpitations (*n* = 13), dizziness (*n* = 8), and weakness (*n* = 9). Distal symptoms included mobility issues (*n* = 25), sleep problems (*n* = 17), concentration difficulties (*n* = 13), and reduced appetite (*n* = 6).

All participants (100%) described how symptoms of TDT fluctuated across their transfusion cycle, reporting that their symptoms were at their worst before their RBCTs (Fig. 1). Most participants regularly experienced fatigue and weakness, the severity of which typically worsened before they underwent an RBCT and lessened afterwards (*n* = 23/29 and *n* = 8/9, respectively). All other symptoms mainly occurred before they underwent an RBCT.

### TDT treatment

Most participants reported receiving an RBCT every 3 to 4 weeks (*n* = 23). For most participants (*n* = 19), the duration of each appointment for an RBCT varied from 3 to 10 h. Twenty-nine participants reported taking ICT; most administered it orally (*n* = 25) and four received it subcutaneously. The frequency of administration ranged from one to three times a day for oral chelators and three daily injections to one every 5 to 7 days for subcutaneous administration.

### HRQoL impacts

Participants reported that the symptoms of TDT, alongside RBCTs and ICT, impacted their daily life and HRQoL. TDT’s impacts on HRQoL fluctuated across their transfusion cycle and were worse before RBCTs. For example, 29 participants reported that TDT impacted their ability to carry out daily activities, including household chores (*n* = 21), leisure activities (*n* = 21), work and school (*n* = 29), and social activities (*n* = 23). These 29 participants further described how the impacts of TDT fluctuated across their RBCT cycle, reporting that they avoided most activities before but not after an RBCT.

### Key dimensions of health not captured by the EQ-5D-5L DS

**Fig. 2:**
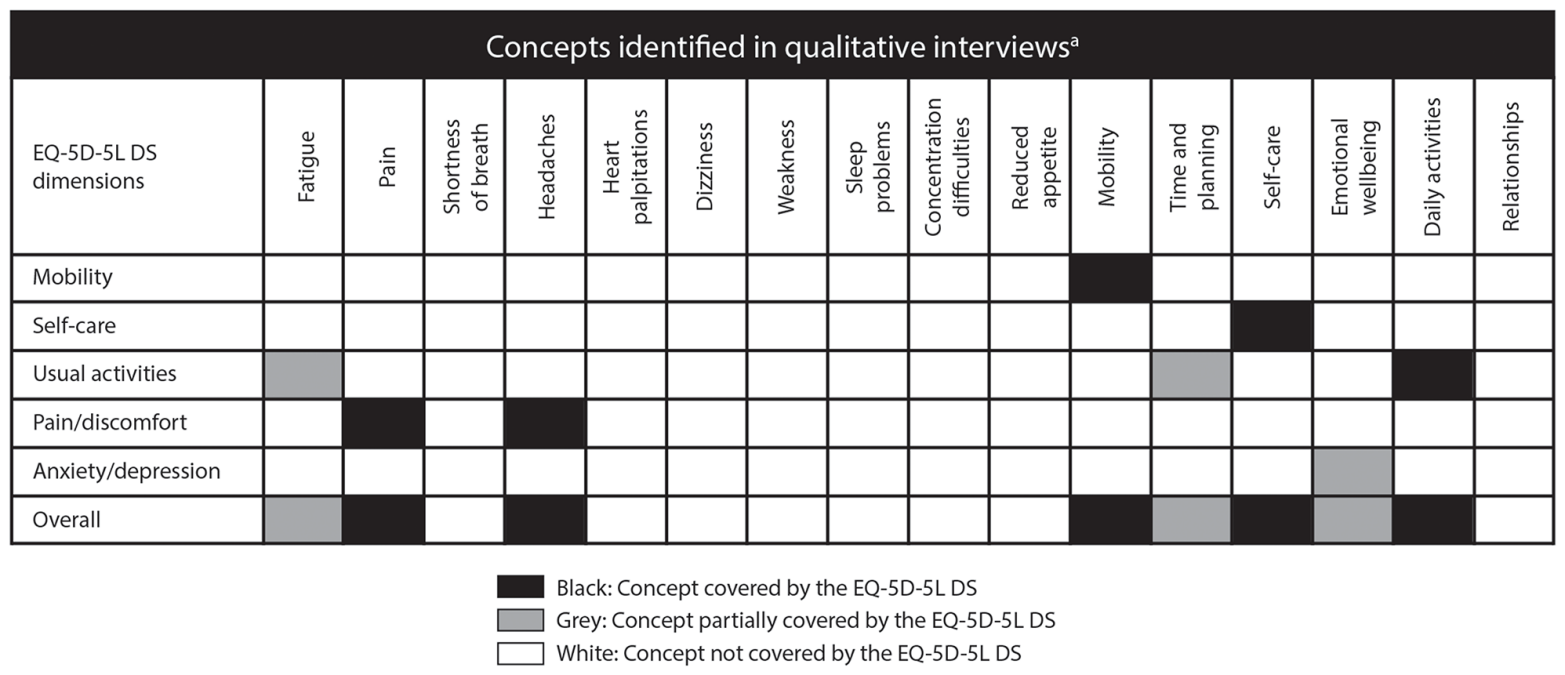
Coverage of identified concepts across the five EQ-5D-5L dimensions*DS* descriptive system

Sixteen HRQoL-relevant concepts relating to the burden of disease in TDT were identified from the interviews, including fatigue, pain, shortness of breath, heart palpitations, headaches, dizziness, weakness, sleep problems, concentration difficulties, reduced appetite, decreased mobility, time and planning, self-care, emotional wellbeing, daily activities, and relationships. Of these 16 concepts, the EQ-5D-5L DS did not cover eight (i.e., shortness of breath, heart palpitations, dizziness, weakness, sleep problems, concentration difficulties, reduced appetite, and relationships) and partially covered three (i.e., fatigue, time and planning, and emotional wellbeing). Thus, the EQ-5D-5L DS did not adequately capture 11 of the 16 (68.8%) concepts elicited across all interviews relating to the symptoms of TDT, the functional issues associated with TDT, and TDT’s impacts on HRQoL (Fig. 2).

### Perceived relevance of the EQ-5D-5L DS in measuring HRQoL

Approximately 42.9% of participants (*n* = 12/28) reported negative (*n* = 7/28 [25.0%]) or neutral (*n* = 5/28 [17.9%]) overall impressions of the EQ-5D-5L DS. Participants who provided reasons for negative/neutral impressions of the EQ-5D-5L DS reported that it was too minimalistic and did not capture the nuances of living with TDT (*n* = 1/28 [3.6%]), such as that it was missing relevant concepts such as social support (*n* = 1/28 [3.6%]) and had a poor layout (*n* = 1/28 [3.6%]):

It’s just very minimalistic, right? Like for example, today I have other things affecting my health, other than thalassemia so it would be kind of inaccurate to say you know, like “Oh yeah, I have problems walking today” because it’s not due to anything thalassemia related… it’s not as nuanced.” – Participant 248, US.

Further, 11 of 24 participants (45.8%) reported that the EQ-5D-5L DS did not adequately capture their experience of living with TDT. Most of these 11 participants claimed that because the EQ-5D-5L used a recall period of “today,” its questions did not capture the fluctuating nature of TDT symptoms based on their RBCT cycle (*n* = 8/24 [33.3%]):

I don’t think it does a very good job of capturing my TDT … if it asked me a day before my transfusion, those answers would be completely different.” – Participant 207, US.

Additionally, most participants (*n* = 20/27 [74.1%]) reported that the EQ-5D-5L DS did not capture important concepts of their experience living with TDT, including symptoms and comorbidities (*n* = 6/27 [22.2%]), such as fatigue (*n* = 3/27 [11.1%]); broader aspects of mental health aside from anxiety/depression (*n* = 4/27 [14.8%]); practical aspects relating to the requirement for RBCTs (*n* = 3/27 [11.1%)]; finances (*n* = 2/27 [7.4%]); and education/work (*n* = 2/27 [7.4%]):

I would say as well like how it affects your relationships as well … and your education … like stuff like that. Because I think they deserve a lot because it does impact your other stuff as well.” – Participant 118, UK.

Exemplary quotes highlighting the missing concepts are presented in Additional File S3.

### Discordance between qualitative and quantitative data on the EQ-5D-5L dimensions

**Fig. 3:**
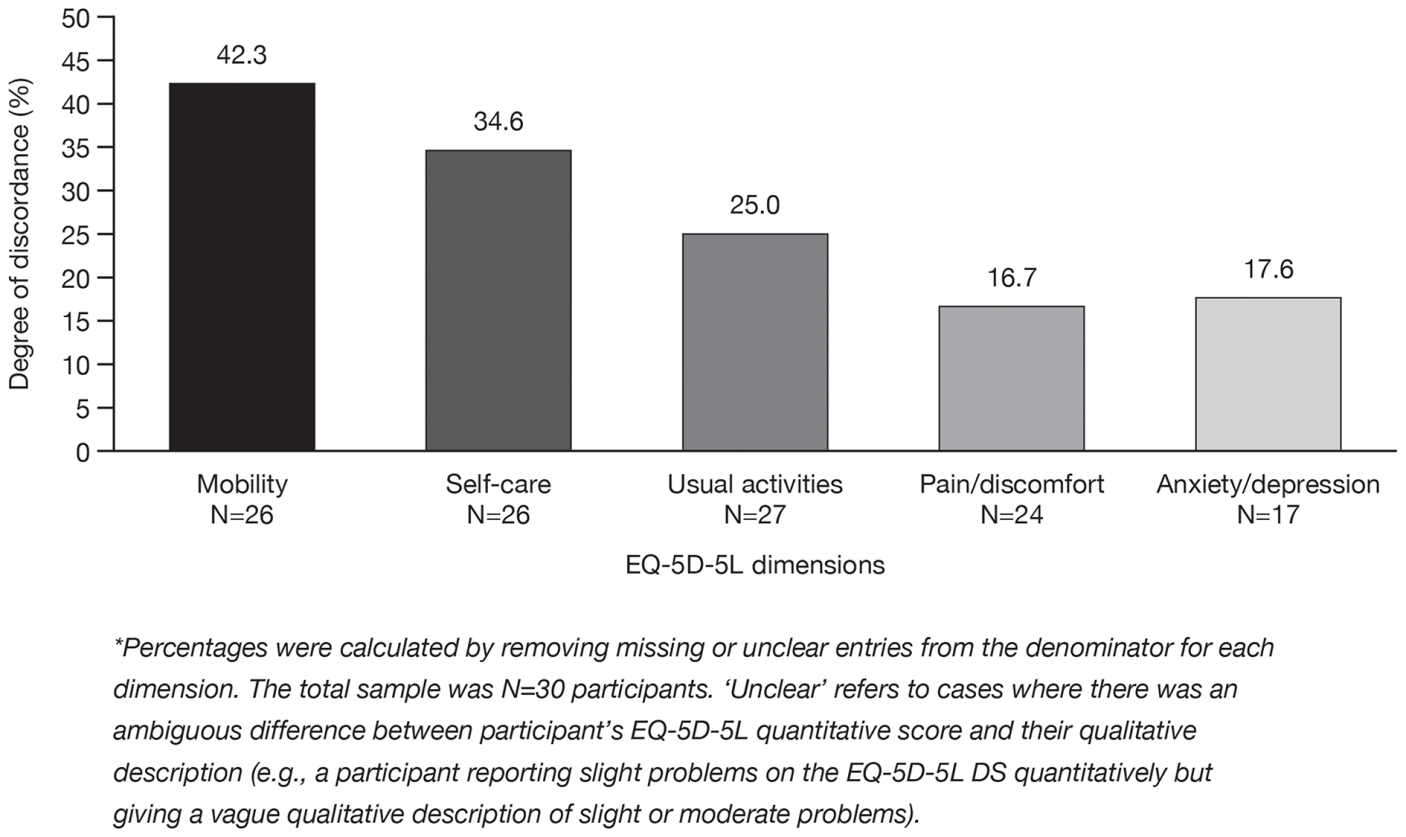
Degree of discordance between participants’ EQ-5D-5L DS quantitative scores and qualitative data. *DS* descriptive system

Some discordances were found between participants’ qualitative and quantitative data for each EQ-5D-5L dimension when participants thought about a time when their TDT was at its worst. The proportion of participants with discordant qualitative and quantitative data ranged from 16.7 to 42.3% across the five EQ-5D-5L dimensions (Fig. 3). In these instances, participants described greater problems with mobility, self-care, usual activities, pain/discomfort, and/or anxiety/depression during their interview compared to their corresponding EQ-5D-5L DS score. Exemplary quotes highlighting the degree of discordance observed between participants’ EQ-5D-5L DS quantitative scores and qualitative descriptions are shown in Additional File S4.

## Discussion

Findings from this study highlight the significant impact of TDT on patients’ HRQoL and the limitations of the EQ-5D-5L DS to adequately capture HRQoL in a chronic rare disease population such as TDT. These findings add to the existing literature confirming the substantial humanistic burden and ongoing unmet needs of patients living with TDT [2, 4, 15–17, 31–50].

Participants reported that TDT symptoms typically worsened before an RBCT and improved afterwards, fluctuations which impacted their HRQoL. Specifically, participants reported that they avoided daily activities such as performing household chores, seeing friends and family, and playing sports before RBCTs but not after them due to the positive impacts of this treatment. These insights correspond with previous findings showing that individuals with TDT experience worse pain and fatigue in the 5 days preceding their RBCTs [44]. To our knowledge, this study is also the first to illustrate the burden of TDT in a conceptual model, highlighting how the various TDT symptoms and impacts relate to one another and how a potential improvement or worsening in one area may have a knock-on effect on other symptoms and areas of life.

Our findings also question the content validity of the EQ-5D-5L DS and the ability of the derived health utility index score to fully represent TDT’s impacts on HRQoL. Notably, several key HRQoL concepts of TDT, such as fatigue, were not captured by the EQ-5D-5L DS, as evidenced in the concept mapping results. These findings suggest that the EQ-5D-5L DS may underestimate the humanistic burden of TDT, especially in instances where the concept (e.g., fatigue) may be indirectly captured only (e.g., via fatigue’s potential impact on usual activities). This is supported by multiple studies using the Short Form 36 Health Survey Questionnaire (SF-36) to measure HRQoL, that found individuals with TDT scored the lowest on certain subscales, such as mental health and vitality, which are not covered by the EQ-5D-5L DS [58–62]. Thus, in a clinical trial setting, the unexpectedly high baseline values of the EQ-5D-5L DS may result in a lack of sensitivity to change, potentially limiting the ability of the EQ-5D-5L DS to adequately detect treatment benefit.

The finding that the EQ-5D-5L DS did not capture all relevant concepts is supported by findings from the cognitive debrief of the EQ-5D-5L DS, wherein participants reported that the EQ-5D-5L DS did not capture their experience of living with TDT and missed concepts relevant to their disease experience and HRQoL, such as the negative impact of continual RBCTs on their social support/relationships and fatigue.

Furthermore, a substantial proportion of participants reported negative or neutral overall impressions of the EQ-5D-5L DS, and some participants reported that it failed to adequately capture the fluctuating nature of their TDT since it assesses their health state based on the current day only (i.e., recall period of “today”), an observation that may impact how the EQ-5D-5L DS is administered in a clinical trial setting. Due to the fluctuating nature of TDT as reported by participants, a typical assessment schedule for the EQ-5D-5L DS may not be sufficient to capture the full burden of TDT. These findings may suggest reasons as to why individuals with TDT have been demonstrated to have a high HRQoL in some studies as indicated by EQ-5D-5L index scores [15–17].

Discordance between participants’ qualitative descriptions and quantitative valuations of their EQ-5D-5L DS for times when their TDT was at its worst were also observed. One explanation for this observed phenomenon may be that participants adapted to their disease state over time, given the chronicity of TDT, an observation consistent with HRQoL assessments among individuals living with other chronic diseases such as hemophilia and fibromyalgia [63–65]. This disease-state adaptation (i.e., “disability paradox”) leads patients with TDT to rate their level of problems within the five EQ-5D-5L dimensions as less severe than if it were perceived by individuals in the general population.

### Limitations

Three key limitations of this study should be acknowledged. First, although data saturation was achieved, participants were self-selected, and their experiences may therefore not represent the experiences of all individuals living with TDT. Second, to reduce participant fatigue, not all participants answered the cognitive debriefing questions, which may have resulted in the study not capturing the full range of views among the study sample. Third, as participants were asked to think about a day when their TDT was “at its worst” when completing the EQ-5D-5L DS, there is risk of recall bias.

## Conclusions

Findings from this study highlight the substantial humanistic burden and unmet need associated with living with TDT, with participants reporting a wide range of symptoms and functional impacts that negatively affect their HRQoL. These findings also suggest that the EQ-5D-5L DS may not adequately capture key dimensions of health relevant to the TDT patient experience and that a typical assessment schedule for the EQ-5D-5L DS may not capture the fluctuating nature of the condition, which could result in the derived health utility index scores not sufficiently representing the burden of disease in TDT. Further exploration of the impact of bolt-ons on the sensitivity of the EQ-5D-5L DS to assess HRQoL in the TDT is still needed, alongside the need for researchers to consider the use of event-driven data collection for this instrument. In addition, the need for the development of a TDT-specific instrument could be explored if the EQ-5D-5L is still not considered fit for purpose in this population.

### Electronic supplementary material

Below is the link to the electronic supplementary material.

Supplementary Material 1

Supplementary Material 2

Supplementary Material 3

Supplementary Material 4

## Data Availability

No datasets were generated or analysed during the current study.

## Abbreviations

EQ-5D-5L: EuroQol 5 Dimensions 5 Levels
DS: Descriptive system
HRQoL: Health-related quality of life
ICT: Iron chelation therapy
RBC: Red blood cell
RBCT: Red blood cell transfusion
SD: Standard deviation
TDT: Transfusion-dependent β-thalassemia
