# Supplementary material for: Appropriateness of the EQ-5D-5L in capturing health-related quality of life in individuals with transfusion-dependent β-thalassemia: a mixed methods study
Source: Health Qual Life Outcomes. 2024 Jul 11;22:54. doi: 10.1186/s12955-024-02265-8 (PMC11241824; doi:10.1186/s12955-024-02265-8)
Supplement: Supplementary file 1 — Supplementary Material 1 [file 12955_2024_2265_MOESM1_ESM.docx]

Additional File 1 Saturation matrices of TDT symptoms (Saturation Matrix 1) and impacts on HRQoL (Saturation Matrix 2) identified from the qualitative interviews.

Saturation Matrix 1: TDT symptoms

|  | UK101 | UK102 | US202 | US204 | US201 | UK110 | US205 | UK111 | UK106 | UK116 | US207 | UK118 | UK107 | FR320 | FR319 | US210 | UK120 | US228 | US248 | US250 | US252 | FR333 | FR332 | US245 | US233 | UK128 | UK129 | UK130 | US253 | US254 |
| --- | --- | --- | --- | --- | --- | --- | --- | --- | --- | --- | --- | --- | --- | --- | --- | --- | --- | --- | --- | --- | --- | --- | --- | --- | --- | --- | --- | --- | --- | --- |
| Fatigue | S | S |  | S | S | S | S | S | S | S | S | S | S | S | S | S | S | S | S | S | S | S | S | S | S | S | S | S | S | S |
| Pain | P | S | S | P | S | S | P | S | S | S | P | P | S | S | S | S | S | S | P | P | S | S |  |  |  | P | S | S | S | S |
| Shortness of breath | S | S | S | S |  | S | S | S |  | S |  | S |  | S | S | S |  |  | S |  |  | S | S | S |  |  |  |  | S |  |
| Headaches |  | S | S | S | S |  |  |  |  | S | S |  |  | P | S | S |  | S | S | S |  | S | S |  |  | S | S | S |  |  |
| Heart palpitations |  | P | S | S |  | P | P | P |  |  |  | S |  |  | S | S |  |  |  | S |  | S | S |  |  |  | S |  |  |  |
| Dizziness |  |  |  | S |  | S | S |  |  |  |  |  |  | P |  | S |  |  | S |  |  | S |  |  |  | S |  |  |  |  |
| Weakness | S |  |  |  |  |  |  | S |  |  | S | S |  | S | S |  |  | S |  |  |  | S | P |  |  |  |  |  |  |  |
| Sleep | S |  | S |  | S | S | S |  |  |  | S |  | S | S | P | S | S |  |  |  | S | S | S |  |  | S |  | S |  | S |
| Concentration difficulties | S |  |  | S |  | S | S | P |  | P |  | S |  |  | S |  |  |  |  |  |  | S |  | S |  | S | S | S |  |  |
| Reduced appetite | S |  |  |  | S | S |  |  |  |  |  |  |  | S | P |  |  |  |  |  |  |  |  | S |  |  |  |  |  |  |
| Mobility issues | S | S |  | S |  | P | P | S |  | S |  | P |  | S | S | P | P | S | P | P | P | S | P | S | P | P | S | P | S | S |

The columns specify interviews in order of completion. Grey cells indicate the point at which a concept was first spontaneously mentioned.
*HRQoL* health-related quality of life; *P* probed; *S* spontaneously reported; *TDT* transfusion-dependent β-thalassemia

Saturation Matrix 2: TDT impacts on HRQoL

|  | UK101 | UK102 | US202 | US204 | US201 | UK110 | US205 | UK111 | UK106 | UK116 | US207 | UK118 | UK107 | FR320 | FR319 | US210 | UK120 | US228 | US248 | US250 | US252 | FR333 | FR332 | US245 | US233 | UK128 | UK129 | UK130 | US253 | US254 |
| --- | --- | --- | --- | --- | --- | --- | --- | --- | --- | --- | --- | --- | --- | --- | --- | --- | --- | --- | --- | --- | --- | --- | --- | --- | --- | --- | --- | --- | --- | --- |
| **Time and planning** | P | S | S | S | S | P | S | S | S | S | S | S | S | S | S | S |  | S |  | S | S | S |  | S | S | S |  | S | S | S |
| **Self-care** |  | S |  | P |  | S | P | S |  | P | P | P |  | S | P | S |  | P |  | P |  | S |  |  |  | S |  | S |  | P |
| **Emotional wellbeing** |  |  |  |  |  |  |  |  |  |  |  |  |  |  |  |  |  |  |  |  |  |  |  |  |  |  |  |  |  |  |
| Overall mental health | S | S | S | S | P | S | S | S | P | S | S | S | S | S | S | S | S | S | S | P |  | S | S | S | P | S | S | S | P | S |
| Anxiety and depression |  | S | S | S | S | P | S | S |  | S | S |  | S | S | S | S | S | P | S | P |  | P | P | P | P | S | P | S |  | S |
| **Daily activities** |  |  |  |  |  |  |  |  |  |  |  |  |  |  |  |  |  |  |  |  |  |  |  |  |  |  |  |  |  |  |
| Household | S | S |  | S | S | P | S | S |  | P |  | S |  | S | P | S | P | P |  | P | S | S |  | S | P |  | P | S |  |  |
| Leisure | S | S | S |  | S | S | S | P |  | P | P | S | S | S | S |  | S | S | P | P | P | P |  | S | S |  | S | S | S | S |
| Social | S | S | S | S | S | S | S | P | S | S | S |  | S | S | S | S | S | S | S | S | S | S |  |  | S |  | S | S | S | S |
| Work/school | S | S | S | S | S | S | P | P | S | S | S | S | S | P | P | S | S | S | S | P | S | S | S | S | S |  | S | S | S | S |
| **Relationships** | S | S | S |  | S |  | S | S |  | S | S |  | S | S | S | P | S |  | S | P | S | S | P | S | S | S | P | S | S | S |

The columns specify interviews in order of completion. Grey cells indicate the point at which a concept was first spontaneously mentioned.
*HRQoL* health-related quality of life; *P* probed; *S* spontaneously reported; *TDT* transfusion-dependent β-thalassemia
