# Supplementary material for: Appropriateness of the EQ-5D-5L in capturing health-related quality of life in individuals with transfusion-dependent β-thalassemia: a mixed methods study
Source: Health Qual Life Outcomes. 2024 Jul 11;22:54. doi: 10.1186/s12955-024-02265-8 (PMC11241824; doi:10.1186/s12955-024-02265-8)
Supplement: Supplementary file 2 — Supplementary Material 2 [file 12955_2024_2265_MOESM2_ESM.docx]

**Additional File 2** Detailed qualitative analysis findings of TDT symptoms and impacts on HRQoL.

**Symptoms**

***Fatigue***

**Fatigue was described as having** no or low energy (*n* = 14/29) and/or not feeling refreshed after resting (*n* = 6/29). The majority of participants described having worse fatigue before RBCTs (*n* = 23/29) and more energy just after them (*n* = 24/29):

“On 18th I’ve got transfusion so the 19th will be my day of happiness, I will feel my best, I can go to Mount Everest, I can do anything that day! I will [be] full of energy. But then as soon as I go near to my transfusion time, I feel like “oh my God”, it’s just, waking up from the bed and just getting dressed up or anything, it’s hard.” – Participant 102, UK

***Weakness***

Nine participants reported experiencing weakness, described as feeling fragile (*n* = 1/9) or having weak and heavy arms, legs, or body (*n* = 5/9):

*“I think there’s just days when I am extra tired or feeling weak, and that has a lot to do with… most of the time like… when I’m due for a transfusion… the week before is just a real struggle.” – Participant 207, US*

***Pain***

Twenty-seven participants reported experiencing pain, including aching (*n* = 6/27), throbbing (*n* = 4/27), cramping (*n* = 4/27), and stabbing (*n* = 3/27), which were located mostly in the back or spine (*n* = 22/27):

“I get a lot of bone pain... generally right before my transfusion is due. A lot of bone pain where it’s like throbbing and just very painful.”
 – Participant 253, US

*“You can get some pain in your lower back, and that’s normally when you’re sort of really low on blood, you tend to feel some lower back pain which [is] normally, as I said, three or four days before your blood transfusion.” – Participant 107, UK*

***Shortness of breath***

Seventeen participants reported experiencing shortness of breath, especially when physically active, such as when climbing stairs:

“When I get closer to my transfusion date, even the little task gets hard to do if I’m… out of breath.” – Participant 204, US

***Headaches***

Seventeen participants reported experiencing headaches, described as bad (*n* = 3/17) and intense (*n* = 1/17):

“Before my blood transfusions I’m usually really down. And it’s like probably like most times I’m just down like and fatigued where I’ve got a really bad headache.” – Participant 128, UK

***Heart palpitations***

Thirteen participants reported experiencing heart palpitations, described as a racing or pounding heart (*n* = 3/13) or heartbeats in the head (*n* = 1/13):

*“[When I’m feeling at my worst] I’m going to be very tired...I’m going to have tachycardia, palpitations, even at night, not doing anything specific [laughs], just sleeping, I’m going to have a pounding heart...I’m going to be walking, it’s going to be [sigh], even walking a little.” – Participant 319, France*

***Dizziness***

Eight participants reported experiencing dizziness, described as feeling like the room is spinning (*n* = 3/8), leading them to be at risk of falling over (*n* = 2/8), and causing nausea and vomiting (*n* = 3/8):

*“At my worst, when I feel dizzy, it’s nauseating, like it feels like I need to hold still and not move because you know, the room is spinning.” – Participant 204, US*

***Problems with mobility***

Twenty-five participants reported experiencing mobility difficulties. Most mentioned walking at a slower pace or, before undergoing an RBCT, being unable to walk long distances without regular breaks (*n* = 20/25) due to experiencing pain (*n* = 9/20), fatigue (*n* = 6/20), and breathlessness (*n* = 4/20). Four participants reported being unable to walk in these periods due to the severity of these symptoms:

“During the week prior to my transfusion, if I walk for a little while or not even for a long time at all, it feels like my calves are becoming tetanised, or if I climb up the stairs, I’m going to feel like my feet weigh, I don’t know, 500kg.” – Participant 333, France

***Problems with sleep***

Seventeen participants mentioned experiencing sleep problems, including daytime sleepiness and the need for naps (*n* = 11/17) and/or difficulty falling or staying asleep (*n* = 8/17) due to pain (*n* = 6/8) or anxiety and restlessness (*n* = 1/8):

“Worst would be on the third week after my transfusion, so the week before my transfusion, so the week before my transfusion I would feel like … I’m not able to sleep much or if I’m sleeping obviously all day [pause] and just don’t feel the energy.” – Participant 101, UK

***Difficulty concentrating***

Thirteen participants described having difficulty concentrating on tasks, such as cooking, work, and processing information. Eight participants attributed this difficulty to severe fatigue:

*“Trying not to sort of doze off maybe… tune out of a meeting, at work, that becomes increasingly more difficult… when I am, you know when I’m in the few days leading up to a transfusion.” – Participant 130, UK*

***Reduced appetite***

Six participants reported not eating at all or eating smaller portions due to fatigue (*n* = 2/6) and nausea (*n* = 2/6) affecting their appetite:

*“I mean lack of appetite, I think it would be just a few days before the transfusion, I wouldn't feel like eating anything, it would be small portions or nothing at all.”
– Participant 101, UK*

**HRQoL impacts**

***Time and planning***

Twenty-three participants noted TDT’s impact on their time, causing them to have to juggle multiple medical appointments and manage their medical care around other activities:

“Being able to juggle like my job and my social life and my healthcare, like it’s like a juggling act to be honest, like making sure everything works perfectly with my schedule and making sure that I’m feeling up to doing all the activities that I like to do.” – Participant 201, US

*“When I’m not run down and I’m able to sort of, you know, do my day-to-day... make sure I’m on top of my medication and… I have, like a good routine, I guess. But it’s very difficult to stick to those routines because when you’re not well, you just get off that sort of routine. And I personally, I like to… stick to my routine but it’s extremely difficult with thalassaemia.”
– Participant 128, UK*

***Daily activities***

Twenty-nine participants reported that TDT impacted their ability to carry out daily activities, including household chores (*n* = 21/29), leisure activities (*n* = 21/29), work and school (*n*= 29/29), and social activities (*n* = 23/29).

All participants who reported impacts to daily activities (*n* = 29) described the influence of the RBCT cycle. Most participants reported avoiding carrying out household chores (*n* = 14/29), leisure activities such as going on family outings and playing sports (*n* = 13/29), and seeing friends and family (*n* = 20/29) just before they received an RBCT. Participants also mentioned struggling with (*n* = 19/29) or being prevented from attending (*n* = 3/29) work and school during these periods. In contrast, after undergoing an RBCT, participants reported no or very limited difficulty performing daily activities:

*“As I got closer and closer to a transfusion, I’d see my friends less and less because I just didn’t really want to have a conversation with anyone or really engage in anything because of… obviously I was tired and everything. But I was also feeling a little bit miserable and a little bit sorry for myself that my legs were aching, and I was tired.” – Participant 130, UK*

*“The week before [my] transfusion, when I’m around doing like helping around the house… doing dishes because I get sick of laying in bed and I want to do something, and I go down to wash the dishes, I have to put a chair behind myself because my heart starts racing and I get shortness of breath. So, I have to sit down in intervals and then sometimes I even give up and I just come back to bed.” – Participant 210, US*

***Self-care***

Seventeen participants reported that TDT impacted their ability to self-care and that this ability depended on where they were in their RBCT cycle, with some participants feeling the least independent before RBCTs (*n* = 7/17):

*“But then at the time [the period before RBC transfusion] I ask my mum, “Can you wash my hair for me? Can you like wash my back and stuff”. And then sometimes I can’t fasten like zips on my clothes at the back or my bra strap, so my mum or sister have to do it.”
 – Participant 116, UK*

***Emotional wellbeing***

Twenty-nine participants reported that TDT impacted their emotional wellbeing. Most participants reported experiencing anxiety (*n* = 16/29) or depression (*n* = 17/29), including having suicidal thoughts (*n* = 2/29):

“I definitely feel depressed on the days of my transfusion because I usually go alone …it impacts your emotions, and you are just reminded, like this is your life, and you have to do this every month and it’s a lot.”
 – Participant 205, US

***Relationships***

Fifteen participants reported that TDT impacted their relationships. Some participants reported that their family and friends were not supportive because they lacked an understanding of the constraints of TDT (*n* = 5/15), and others reported concerns about forming romantic relationships (*n* = 5/15):

*“Missing out on family time sometimes, you know, being tired, more tired than normal and having to have early nights, not being able to spend time with my wife, sometimes… has an impact. So, all those things really kinda impact me.”
 – Participant 107, UK*
