# Supplementary material for: Appropriateness of the EQ-5D-5L in capturing health-related quality of life in individuals with transfusion-dependent β-thalassemia: a mixed methods study
Source: Health Qual Life Outcomes. 2024 Jul 11;22:54. doi: 10.1186/s12955-024-02265-8 (PMC11241824; doi:10.1186/s12955-024-02265-8)
Supplement: Supplementary file 3 — Supplementary Material 3 [file 12955_2024_2265_MOESM3_ESM.docx]

**Additional File 3** Exemplary quotes depicting TDT concepts perceived to not be captured by the EQ-5D-5L DS.

| **Concept** | **Example quotes** |
| --- | --- |
| **Fluctuating nature of TDT** | *“I don’t think that it’s a good [instrument], it’s one day that I just happen to be feeling good, maybe it’s a bad day, I mean I don’t know how you guys rank or rate these things but… I think for somebody that gets, for transfusion-dependent, you know if you catch somebody on a day right before they get transfusion, they’re not going to feel good, like it’s going to skew your results, but I don’t know you guys…rate it and there’s no asking like, “When was your last transfusion?” I think it’s a little…it can be a little misleading.”  – Participant 253, US* |
|  | *“It really is sort of an ever-moving process of feeling good and feeling not so good and you know, doing lots of stuff and then having to crash and save energy and not being able to do lots of stuff. I think, to try and sum it up in like a sentence say would really, wouldn’t do it justice, wouldn’t do it justice to sort of how variable it is on a day-to-day, week-to-week, month-to-month, year-to-year basis.” – Participant 130, UK* |
| **Fatigue** | *“Erm, fatigue has not been touched on that much, maybe how much, how well we have slept in the night.”  – Participant 210, US* |
| **Social support** | *“It would be good to have a question on how… what social support do you have because I feel like just from my own experience and you know, learning about it from other people who’ve lived with thalassemia, a few of my friends, the social support is a huge aspect on you know, how a person feels about the condition or what they can do.” – Participant 252, US* |
| **Relationships** | *“The impacts it has on my relationships… it’s kind of a thing that I hadn’t really even thought about… we go through it where I talk to husband about it and stuff, but like when you say how does it impact those relationships, like I’d never really thought about it until you had said that. Like yeah it actually does impact them more than I, you know I think about, ‘cos in those moments I think about myself, right, I’m very like oh I’m tired, I don’t wanna deal with it. But I’m not thinking about how it affects them… that’s something that also could be captured on this survey.” – Participant 207, US* |
| **Education/work** | *“So, there you go. Your boss agrees with you, but at the same time sometimes there are little sentences, where you are told “The fact that you are not here has an impact” so... well I’ll skip the details, but... I think... it’s pretty sure it has had an impact on my career path. Well, just my whole life path, whether it’s professional, personal, that’s it.” – Participant 319, France* |
| **Practical impacts** | *“Yeah, there were no practical questions i.e., like, “Practically speaking, how does it affect your life?” Or, or kind of you know, explores just the kind of medical things but it doesn’t really look at the practical side.”  – Participant 106, UK* |
| **Finance** | *“And stuff like finance and stuff and you’re fine because financially it does sometimes, because if you don’t drive, stuff like public transport as well, it’s not always…Yeah, so financially it depends as well, so stuff like that I would say.” – Participant 118, UK* |
| **Broader aspects of mental health** | *“I guess anxiety and depression is good but like I just feel like it would be good to have a bit more, more options for like … the social aspect of it or… do you want to, like we just discussed, do you want to go out and do leisure activities or things that you don’t, like you don’t want to or like how do you actually feel today like about living with this condition? I don’t know if anxiety and depression like covers all of the mental health issue that there is I guess for any patient.” – Participant 252, US* |

*DS* descriptive system; *TDT* transfusion-dependent β-thalassemia
