# Supplementary material for: Appropriateness of the EQ-5D-5L in capturing health-related quality of life in individuals with transfusion-dependent β-thalassemia: a mixed methods study
Source: Health Qual Life Outcomes. 2024 Jul 11;22:54. doi: 10.1186/s12955-024-02265-8 (PMC11241824; doi:10.1186/s12955-024-02265-8)
Supplement: Supplementary file 4 — Supplementary Material 4 [file 12955_2024_2265_MOESM4_ESM.docx]

**Additional File 4** Exemplary quotes depicting discordance between EQ-5D-5L DS scores and qualitative data.

| **Dimension** | **Example quotes** |
| --- | --- |
| **Mobility** | *“Sometimes all I can do is just like lay in bed or just take it easy, just rest? That’s kind of all I can manage to do”  – Participant 204, US*  **Reported only moderate problems with mobility in the EQ-5D-5L DS** |
|  | *“Out of breath, that’s it, there you go, and actually, I can’t do it, I tell myself that I can’t stand straight. For example, if I walk, I walk 3 meters, it’s done, I can do it no more, I have to rest somewhere, I have to sit down.”  – Participant 332, France*  **Reported only slight problems with mobility in the EQ-5D-5L DS** |
| **Self-care** | *“Sometimes when I’m tired, I’m going to put a little shower stool in my bathtub, so I could stand but I’m actually so tired that I sit down.”* *– Participant 333, France*  *“I would say self-care on bad days it’s harder to get ready for the day erm when you’re feeling really tired or you’re feeling depressed because of it. It’s really like such a chore to even like get ready for the day, you feel so tired. So, it does impact self-care.”– Participant 205, US*  **Both reported no problems with self-care in the EQ-5D-5L DS** |
| **Usual activities** | *“On the worst days I can’t go out with my friends, and I can’t do stuff because I’m very tired.” – Participant 116, UK*  *“The days before transfusion, I am really tired and I may not wanna cook dinner or we’ll just order takeout or, … maybe we won’t go out, you know as a family or whatever and do those activities just because I am tired.” – Participant 207, US*  **Both reported no problems with usual activities in the EQ-5D-5L DS** |
| **Pain/discomfort** | *“I do get like lower back pain and headaches... I’ll usually go for transfusion every four weeks, but because of scheduling issues or whatnot, maybe it runs a little bit over those four weeks and then I really do start to feel, I get headaches and lower back pain, and like also leg cramps.” – Participant 207, US*  **Reported having no pain/discomfort in the EQ-5D-5L DS**  *“Quite bad. It’s quite a lot, pretty unbearable, do you want me to give like a number or something?  Maybe about 8 [out of 10].” – Participant 120, UK*  **Reported having moderate pain/discomfort in the EQ-5D-5L DS** |
| **Anxiety/depression** | *“When you’re feeling at your worst, you tend to, as I said, you sleep more, you’re sort of less energetic, you sort of you know, feel down in the dumps as such, you sometimes question you know, why do I have to go through with this and you can sometimes get yourself a little bit worked up.”* – *Participant 107, UK*  **Reported having no anxiety/depression in the EQ-5D-5L DS**  *“Well emotionally I’m very down on those days, I feel very anxious, er, I hate being in bed all day, and then I have to go to sleep in bed for the whole night, it just makes me very anxious. And I just look at my to-do list I’ve wrote, and nothing has gotten done.” – Participant 210, US*  **Reported having slight anxiety/depression in the EQ-5D-5L DS** |

*DS* descriptive system
